# Factors That Influence Undergraduate Medical Students to Choose General Surgery as a Future Career in Saudi Arabia

**DOI:** 10.7759/cureus.48888

**Published:** 2023-11-16

**Authors:** Loai S Albinsaad, Abdullah F Almulhim, Abdullah Albadran, Mohammed Alkhars, Amar A Alonazi, Shima Al Boesa, Majed M Almajed, fatimah A Alhamad

**Affiliations:** 1 College of Medicine, King Faisal University, Al Hofuf, SAU; 2 College of Medicine, King Faisal University, Al Ahsa, SAU; 3 College of Medicine, King Faisal University, Dhahran, SAU; 4 College of Medicine, King Faisal University, Dammam, SAU

**Keywords:** kingdom of saudi arabia (ksa), undergraduates, perception, general surgery, medical students

## Abstract

Objective

The objective of this cross-sectional study conducted in Saudi Arabia was to determine the factors that influence Saudi medical students to choose general surgery as their future specialty and pursue it as a career.

Study design

The study was conducted over a six-month period from December 2022 to May 2023 and employed a cross-sectional design.

Patients and methods

Undergraduate medical students from various universities in Saudi Arabia were included as study participants. Only those who met the study's criteria completed a questionnaire, and the data collected was analyzed using IBM Corp. Released 2019. IBM SPSS Statistics for Windows, Version 26.0. Armonk, NY: IBM Corp.

Results

Out of the 283 medical students included in the study, 53% were females, and 55.1% were junior students. The results revealed that the most common influential factors towards selecting a general surgical specialty were "Opportunity to be involved in patient care" (86.9%), "Opportunities for higher studies or further specialization" (85.9%), and "Compatible with personality" (84.8%).

Conclusion

This study concluded that several factors moderately influenced medical students considering general surgery as their future career. Notably, female students were found to be more likely to be influenced by research opportunities, less stressful working conditions, shorter working hours, and having family members in the same specialty.

## Introduction

For undergraduate medical students, choosing a future medical specialization is a crucial and frequently difficult choice [1]. There are many different specializations available in the medical field, and in recent years, this range of option has become increasingly competitive and difficult. These factors encompass elements such as gender and societal status, lifestyle considerations, anticipated income prospects, the allure of specific working hours and on-call duties, the specialty’s reputation, and the geographical availability of residency programs [2,3]. It is noteworthy that, unfortunately, some students make their career decisions based more on the practical consideration of residency program accessibility than on their intrinsic passion or personal preferences [3]. In this complex field, general surgery maintains a prominent and long-standing role as one of the primary specialties in medicine. The general surgeon continues to play an essential role in the operation of any healthcare facility [4]. However, recent empirical investigations have unveiled a disconcerting trend-an alarming decline in the selection of General Surgery as a future career path among medical students [5,6]. To illustrate, a comprehensive study conducted in the United States starkly revealed a consistent annual 5% reduction in students’ interest in pursuing general surgery during their four-year tenure in medical college [5]. Similarly, across the Atlantic, an Irish study documented that more than two-thirds of the participating students did not entertain the notion of general surgery as their forthcoming specialty. The rationale behind this downward trajectory is manifold, encompassing the formidable challenges posed by maintaining a harmonious work-life balance and the demanding nature of the specialty’s working hours [6]. In striking contrast to these international trends, studies conducted in Saudi Arabia have illuminated a palpable proclivity among students to embrace general surgery as their prospective career path. A study undertaken in Abha, Saudi Arabia, reported that approximately 26% of medical students preferred general surgery, catapulting it to the summit of their career aspirations [7]. Similarly, in Riyadh, the capital city of Saudi Arabia, an additional study underscored this inclination, with 27% of students opting for general surgery as their future specialization [8]. This growing trend and increased interest in general surgery as a future career among Saudi medical students begs a compelling question, given the stark differences in their choices when compared to their international counterparts. Our research project centers around this fascinating paradox as we seek to understand the distinct and contextual elements that influence career decisions in various geographic areas. This research essentially begins a thorough investigation of the complex factors that influence undergraduate medical students' decisions when they are thinking about pursuing a general surgery career. Finding out what factors affect Saudi medical students' decision to specialize in general surgery and pursue it as a career is the aim of this study.

## Materials and methods

This study utilized a cross-sectional design to investigate factors related to choosing a general surgery specialty among undergraduate medical students in Saudi Arabia. The study was conducted over a period of six months, from December 2022 to May 2023, and included participants from various universities in the country. The study population consisted of undergraduate medical students, while postgraduates were excluded from the study. Ethical approval was obtained from the institutional review board of the College of Medicine, King Faisal University, on December 20, 2022. Before their participation, all participants signed an informed consent form in either Arabic or English. Throughout the study, ethical considerations and confidentiality of participants' information were strictly maintained through the use of anonymized data.

Sample size and technique

To determine the required sample size, a calculation was performed, indicating that a total of 385 participants from different universities in Saudi Arabia would be needed. This calculation was based on a confidence interval of 95% and a margin of error of 5% [9]. Once informed consent was obtained, participants who met the study's criteria completed a questionnaire consisting of two parts: (i) sociodemographic data and (ii) 22 items exploring factors related to choosing general surgery as a specialty. 

Statistical analysis 

The collected data were analyzed using IBM Corp. Released 2019. IBM SPSS Statistics for Windows, Version 26.0. Armonk, NY: IBM Corp., developed by IBM Corporation, USA. Descriptive analysis was conducted, with metric variables presented as mean ± standard deviation and categorical variables presented as numbers and percentages. To facilitate the analysis, medical students were categorized into two groups: junior students (second year, third year, and fourth year) and senior students (fifth year, sixth year, and medical interns). Between-group comparisons were performed using the Chi-square test. Statistical significance was determined by a p-value of less than 0.05.

## Results

The response rate was 125.9%, which was higher than expected. Our sample size was 385, yet we collected data from 485 medical students, of whom 202 were excluded as they did not express an interest in pursuing a general surgery specialty. The remaining 283 questionnaires formed our sample population, resulting in an overall response rate of 58.4%. Table 1 presents the basic demographic characteristics of medical students. 26.1% were enrolled in the fourth-year level, with more than half (53%) being female. In addition, 24.4% were studying at Umm Al-Qura University (Table 1). 

**Table 1:** Medical students’ basic demographic data (n=283)

Study data	N (%)
Academic year level	
Second year	36 (12.7%)
Third year	46 (16.3%)
Fourth year	74 (26.1%)
Fifth year	50 (17.7%)
Sixth year	61 (21.6%)
Medical intern	16 (05.7%)
Gender	
Male	133 (47.0%)
Female	150 (53.0%)
University	
Umm AlQura University‎	69 (24.4%)
Majmaah University	45 (15.9%)
King Faisal University	40 (14.1%)
University of Jeddah	19 (06.7%)
Imam Abdulrahman Bin Faisal University	16 (05.7%)
Ibn Sina National College	16 (05.7%)
KSAU-HS	13 (04.6%)
Jazan University	10 (03.5%)
Al Jouf University	09 (03.2%)
Almaarefa University	08 (02.8%)
Al Baha University	06 (02.1%)
King Abdulaziz University	05 (01.8%)
Tabuk University	04 (01.4%)
Others	23 (08.1%)

The top most common reasons for choosing general surgery as a future career, according to medical students’ perceptions, were “opportunity to be involved in patient care” (86.9%), “opportunities for higher studies or further specialization” (85.9%), “compatibility with personality” (84.8%), “professionally challenging” (83%), and “sense of calling” (71%), while the least common reason was “family member in the same specialty” (25.8%). When comparing genders, it was observed that female medical students were more likely to choose the general surgery specialty due to the opportunity to do research (p=0.004), less stressful working conditions (p=0.008), shorter working hours (p=0.047), and family members in the same specialty (p=0.011), while male students were significantly more likely to choose the social position status of the field (p=0.045) (Table 2).

**Table 2:** Gender comparison for choosing a general surgery specialty based on influencing factors (n=283) § P-value has been calculated using the Chi-square test. ** Significant at p<0.05 level

Influencing factors	Overall N (%) ^(n=283)^	Male Yes (%) ^(n=133)^	Female Yes (%) ^(n=150)^	P-value ^§^
Opportunity to be involved in patient care	246 (86.9%)	116 (87.2%)	130 (86.7%)	0.891
Opportunities for higher studies or further specialization	243 (85.9%)	114 (85.7%)	129 (86.0%)	0.945
Compatible with personality	240 (84.8%)	111 (83.5%)	129 (86.0%)	0.552
Professionally challenging	235 (83.0%)	113 (85.0%)	122 (81.3%)	0.417
Sense of calling	201 (71.0%)	93 (69.9%)	108 (72.0%)	0.701
Patient characteristics	196 (69.3%)	96 (72.2%)	100 (66.7%)	0.316
Influences from past experiences (in courses & rotations)	196 (69.3%)	88 (66.2%)	108 (72.0%)	0.288
Social position status of the field	194 (68.6%)	99 (74.4%)	95 (63.3%)	0.045 **
Working environment	192 (67.8%)	85 (63.9%)	107 (71.3%)	0.182
Financial prospects	190 (67.1%)	95 (71.4%)	95 (63.3%)	0.148
Influenced by role models	181 (64.0%)	85 (63.9%)	96 (64.0%)	0.987
Characteristics of team and colleagues	178 (62.9%)	85 (63.9%)	93 (62.0%)	0.740
Market dynamics of the specialty	171 (60.4%)	84 (63.2%)	87 (58.0%)	0.376
The acceptance rate to the program	168 (59.4%)	79 (59.4%)	89 (59.3%)	0.991
Opportunity to teach	164 (58.0%)	77 (57.9%)	87 (58.0%)	0.986
Opportunity to do research	151 (53.4%)	59 (44.4%)	92 (61.3%)	0.004 **
Sufficient time for hobbies and personal interests	138 (48.8%)	61 (45.9%)	77 (51.3%)	0.358
Flexible working hours	90 (31.8%)	35 (26.3%)	55 (36.7%)	0.062
Parental preferences	79 (27.9%)	34 (25.6%)	45 (30.0%)	0.406
Less stressful working conditions	74 (26.1%)	25 (18.8%)	49 (32.7%)	0.008 **
Less duration of work hours	73 (25.8%)	27 (20.3%)	46 (30.7%)	0.047 **
Family members in the same specialty	73 (25.8%)	25 (18.8%)	48 (32.0%)	0.011 **

When comparing the perceptions of junior and senior medical students, it was observed that junior students were more likely to associate with choosing general surgery as a future career. This inclination can be attributed to factors such as the working environment (p = 0.002), sufficient time for hobbies and personal interests (p = 0.009), parental preferences (p = 0.024), less stressful working conditions (p = 0.012), less duration of work hours (p<0.001), and family members in the same specialty (p = 0.003), while senior students were more likely to choose the general surgery specialty due to influences from past experiences (p = 0.009) (Table 3). 

**Table 3:** Assessment of medical students’ perceptions toward the general surgery specialty § P-value has been calculated using the Chi-square test. ‡ P-value has been calculated using an independent sample t-test. ** Significant at p<0.05 level.

Influencing factors	Overall N (%) ^(n=283)^	Junior students N (%) ^(n=156)^	Senior students N (%) ^(n=127)^	P-value ^§^
Opportunity to be involved in patient care	246 (86.9%)	135 (86.5%)	111 (87.4%)	0.830
Opportunities for higher studies or further specialization	243 (85.9%)	130 (83.3%)	113 (89.0%)	0.175
Compatible with personality	240 (84.8%)	131 (84.0%)	109 (85.8%)	0.666
Professionally challenging	235 (83.0%)	129 (82.7%)	106 (83.5%)	0.863
Sense of calling	201 (71.0%)	111 (71.2%)	90 (70.9%)	0.958
Patient characteristics	196 (69.3%)	105 (67.3%)	91 (71.7%)	0.431
Influences from past experiences (in courses & rotations)	196 (69.3%)	98 (62.8%)	98 (77.2%)	0.009 **
Social position status of the field	194 (68.6%)	105 (67.3%)	89 (70.1%)	0.618
Working environment	192 (67.8%)	118 (75.6%)	74 (58.3%)	0.002 **
Financial prospects	190 (67.1%)	107 (68.6%)	83 (65.4%)	0.564
Influenced by role models	181 (64.0%)	103 (66.0%)	78 (61.4%)	0.442
Characteristics of team and colleagues	178 (62.9%)	98 (62.8%)	80 (63.0%)	0.976
Market dynamics of the specialty	171 (60.4%)	99 (63.5%)	72 (56.7%)	0.247
The acceptance rate to the program	168 (59.4%)	92 (59.0%)	76 (59.8%)	0.882
Opportunity to teach	164 (58.0%)	98 (62.8%)	66 (52.0%)	0.066
Opportunity to do research	151 (53.4%)	90 (57.7%)	61 (48.0%)	0.105
Sufficient time for hobbies and personal interests	138 (48.8%)	87 (55.8%)	51 (40.2%)	0.009 **
Flexible working hours	90 (31.8%)	57 (36.5%)	33 (26.0%)	0.058
Parental preferences	79 (27.9%)	52 (33.3%)	27 (21.3%)	0.024 **
Less stressful working conditions	74 (26.1%)	50 (32.1%)	24 (18.9%)	0.012 **
Less duration of work hours	73 (25.8%)	54 (34.6%)	19 (15.0%)	<0.001 **
Family members in the same specialty	73 (25.8%)	51 (32.7%)	22 (17.3%)	0.003 **
Total perception score (mean ± SD)	12.9 ± 4.48	13.5 ± 5.09	12.4 ± 3.54	0.044 **

## Discussion

This study evaluates factors that have a significant influence on medical students' decisions to pursue a career in general surgery. Based on feedback from our participants, the following factors emerged as the most influential in choosing a general surgery specialty: involvement in patient care (86.9%), opportunities for ongoing education (85.9%), compatibility with personal attributes (84.8%), professional challenges (83%), and a sense of calling (71%). These factors collectively represent the top five considerations in selecting a career path within the field of general surgery. These findings may have been observed in a study conducted by Peel et al. (2018) [10]. According to the survey conducted (Appendix), the primary reasons for selecting a general surgery specialty were identified as the scientific challenge and patient interaction, which is consistent with the findings of the study conducted by Avan et al. (2003) [11]. However, a study conducted by Gutiérrez-Cirlos et al. (2019) documented that "special interest," "specialty flexibility," and "anticipated outcome" were the three key factors influencing the decision to pursue a career in general surgery among Mexican medical students [12]. Additionally, the study identified three relevant dimensions, namely personal values that evolve during undergraduate training, career satisfaction, and perception of specialty characteristics, which influenced the choice of specialty [13]. On the other hand, in a study conducted in South Africa [14], 33% of the medical students expressed that the length of training was a great deterrent for not choosing a career in the surgical profession, while “hands-on work” was their great motivation. The authors suggested that the recognized factors could play an important role in improving the frequency of surgeons’ applications in all regions. Junior medical students were more likely to choose a career in general surgery than their senior students [15] counterparts, and the most common reasons that attracted them to choose a career in the general surgery field were related to having enough time for hobbies and social interests, parental preferences, a stress-free environment, adequate working hours duration, and influence by family members who are also in the same field, while the influence from previous experiences was the only factor that significantly influences senior students [16]. This contradicted the report of Acai et al. (2020), wherein medical students’ preference for choosing a career as a general surgeon increased significantly after the sixth year, and the prevalence was higher in males [17]. Moreover, our study data indicates that female medical students are inclined towards selecting a general surgery specialty due to research opportunities, a less stressful environment, reduced working hours, and having family members in the same field [18]. In contrast, male students prioritize the social standing associated with the specialty. However, other influential factors have a comparable impact on both male and female students. In Jordan, the most important factors for female students in choosing the right specialty were “on-call schedule” and a focus on community health [2], while among their male counterparts, the flexibility of the specialty, specialty reputation, and anticipated income were the most relevant factors [19]. A study conducted by Kaliyadan et al. (2015) found that male medical students tend to select a specialty based on their aptitude, financial rewards, and research opportunities [3]. Conversely, a study conducted by Sawan et al. (2023) revealed that male students prioritize factors such as a less competitive field, specialist shortages, and patient diversity [20]. Our strengths include involving various regions and universities and having a response rate that exceeded our expectations. Notwithstanding, certain constraints existed, such as the absence of similar research in our region for comparison and the inability to find a validated survey; thus, we formulated a pilot study to establish our own. Female students were greatly influenced by the specialty's reputation and teaching opportunities, while seniority and gender preference affected the specialty choices they made while still in medical school [21]. Continuous lectures and workshops addressing important factors aimed at medical students are essential to increasing interest in a career in general surgery [22]. Getting students hands-on training could be a good way to counteract factors that could influence their decisions about general surgery.

## Conclusions

Female and junior medical students displayed a greater preference for pursuing general surgery compared to their counterparts. The influential factors that stood out among medical students were opportunities for patient care, continuing education, and professional challenges. 

## Appendices

**Table 4:** Questionnaire

[1] Biographical Data:
Do you agree to participate in the survey?
Yes
No
Year:
Second year
Third year
Fourth year
Fifth year
Sixth year
Medical intern
Gender
Male
Female
University
King Faisal University
Imam Abdulrahman Bin Faisal University
King Saud University
Almaarefa University
Princess Nourah bint Abdulrahman University
Alfaisal University
University of Jeddah
Imam Mohammed Bin Saud University
KSAU-HS
King Abdulaziz University
Fakeeh College of medical sciences
Batterje Medical college
Hail University
Umm Al Qura University
Tabuk University
King Khaled University
AL Qassim University
Are you interested in General Surgery specialty
Yes
No
[2] Why do you choose General Surgery as a future career:
Working environment
Yes
No
Financial prospects
Yes
No
Social position status of the field
Yes
No
Opportunity to be involved in patient care
Yes
No
Patient characteristics
Yes
No
Opportunities for higher studies or further specialization
Yes
No
Less stressful working conditions
Yes
No
(influences from past experiences (in courses & rotations)
Yes
No
Influenced by roles models
Yes
No
Family members in same specialty
Yes
No
Parental preferences
Yes
No
Opportunity to teach
Yes
No
Sufficient time for hobbies and personal interests
Yes
No
Market dynamics of the specialty
Yes
No
The acceptance rate to the program
Yes
No
Characteristics of team and colleagues
Yes
No
Compatible with personality
Yes
No
Sense of calling
Yes
No
